# Risk-Association of Five SNPs in *TOX3/LOC643714* with Breast Cancer in Southern China

**DOI:** 10.3390/ijms15022130

**Published:** 2014-01-29

**Authors:** Xuanqiu He, Guangyu Yao, Fenxia Li, Ming Li, Xuexi Yang

**Affiliations:** 1 The First Clinical College, Southern Medical University, Guangzhou 510515, China; E-Mail: lmsh815@163.com; 2 Breast Center Nanfang Hospital, Southern Medical University, Guangzhou 510515, China; E-Mail: ygy531@163.com; 3 School of Biotechnology, Southern Medical University, Guangzhou 510515, China; E-Mails: lifenxia123@gmail.com (F.L.); mingli2006_2006@126.com (M.L.)

**Keywords:** breast cancer, *TOX3/LOC643714*, single nucleotide polymorphism (SNP), susceptibility

## Abstract

The specific mechanism by which low-risk genetic variants confer breast cancer risk is currently unclear, with contradictory evidence on the role of single nucleotide polymorphisms (SNPs) in *TOX3/LOC643714* as a breast cancer susceptibility locus. Investigations of this locus using a Chinese population may indicate whether the findings initially identified in a European population are generalizable to other populations, and may provide new insight into the role of genetic variants in the etiology of breast cancer. In this case-control study, 623 Chinese female breast cancer patients and 620 cancer-free controls were recruited to investigate the role of five SNPs in *TOX3/LOC643714* (rs8051542, rs12443621, rs3803662, rs4784227, and rs3112612); Linkage disequilibrium (LD) pattern analysis was performed. Additionally, we evaluated how these common SNPs influence the risk of specific types of breast cancer, as defined by estrogen receptor (ER) status, progesterone receptor (PR) status and human epidermal growth factor receptor 2 (HER2) status. Significant associations with breast cancer risk were observed for rs4784227 and rs8051542 with odds ratios (OR) of 1.31 ((95% confidence intervals (CI), 1.10–1.57)) and 1.26 (95% CI, 1.02–1.56), respectively, per T allele. The T-rs8051542 allele was significantly associated with ER-positive and HER2-negative carriers. No significant association existed between rs12443621, rs3803662, and rs3112612 polymorphisms and risk of breast cancer. Our results support the hypothesis that the applicability of a common susceptibility locus must be confirmed among genetically different populations, which may together explain an appreciable fraction of the genetic etiology of breast cancer.

## 1. Introduction

Breast cancer continues to be a major contributor to overall morbidity and mortality among women, accounting for 23% of all cancers in women in 2008 [1], and its incidence continues to increase, particularly in several Asian countries [2]. It is considered a complex disease with a combined effect of genetic and nongenetic etiology [3]. In familial linkage studies, several high-penetrance low-frequency mutations in genes confer increased susceptibility to breast cancer [4], including breast cancer 1 gene *(BRCA1*), *BRCA2*, ataxia-telangiectasia mutated gene (*ATM*), *etc.*; however, these causative mutations explain only approximately 25% of the familial risk [5] and almost 5% of breast cancer incidence. Therefore, low-penetrance high-frequency genes/loci might have significant associations with breast cancer risk and might contribute to the remaining 75% of the risk. Recently, in unselected breast cancer patients, several genome-wide association studies (GWAS) or studies of specific candidate single nucleotide polymorphisms (SNPs) have revealed a number of novel genetic susceptibility variants and loci, including *FGFR2*, *TOX3/LOC643714*, *LSP1*, *MAP3K1*, chromosome 8q24, and *CASP8*, which were independently associated with an increased risk of breast cancer. Most of these studies focused primarily on women of European descent [6–11], and replication studies among Asian populations have had mixed success with approximately half of the identified loci [12–15]. Failure to confirm some risk loci could stem from differences in linkage disequilibrium (LD) patterns between European and Asian populations. Other factors may include population differences that vary by ethnicity, family history, menopausal status and tumor status of estrogen receptor (ER), progesterone receptor (PR), or human epidermal growth factor receptor 2 (HER2).

*TOX3*, a gene of uncertain function containing a tri-nucleotide repeat motif, as well as the hypothetical gene *LOC643714* (http://www.sanger.ac.uk/genetics/CGP/cosmic/) [16], encodes a putative high-mobility-group (HMG) box motif nuclear protein, suggesting that it might act as a transcription factor that may be involved in calcium-dependent transcription [17]. Its protein expression has been suggested to predict breast cancer metastasis to bone [18]. A recent study has provided strong *in vitro* evidence implicating *TOX3* rs4784227 as a functional variant for breast cancers in Asian women [19]; however, it failed to identify other SNPs adjusted by rs4784227 associated with breast cancer risk; moreover, this finding has not been replicated by other studies. The specific mechanisms by which causative variants in *TOX3/LOC643714* affect breast cancer risk remain unclear.

In this breast cancer case-control study among Chinese women, we assessed the association of breast cancer with five SNPs (rs8051542, rs12443621, rs3803662, rs4784227, and rs3112612) in the *TOX3/LOC643714* locus to provide confirmatory replicative results of several studies in multiple populations. As subtypes stratified by receptor status suggest that the heterogeneity of genetic associations might result from different etiologic pathways, we also determined whether the presence of ER+/−, PR+/−, and HER2+/− subtype tumors modifies the association with breast cancer risk. Further investigation of these loci in non-European populations may reveal the generalizability of these initial findings and shed new light onto the biological mechanisms by which genetic variants affect breast cancer etiology.

## 2. Results and Discussion

### 2.1. Subject Characteristics

Baseline characteristics of 623 patients with breast cancer are shown in Table 1. The age of all patients at diagnosis/selection was 48.5 ± 10.0 years (range 22–80 years). Overall, the most common tumor histology type in patients was invasive ductal carcinoma (IDC) accounting for 84.1% (524), compared with 5.6% (35) of other histology types (invasive lobular carcinoma (ILC), mucin-producing carcinomas (Muc) and medullary carcinoma (Medul)) and 10.3% (64) of unknown types. Only six patients (1.0%) presented at stage 0 (carcinoma *in situ*), and stages 1 to 4 accounted for 24.6% (153), 24.7% (154), 30.0% (187), and 9.5% (59) of patients, respectively. The cases consisted of 300 (48.2%) ER-positive tumors and 217 (34.8%) ER-negative tumors, while the remaining 106 (17.0%) were untested; 42.9% of patients had PR-positive tumors and 40.0% had PR-negative tumors, with 17.2% untested; and 36.9% of patients had HER2-positive tumors and 41.7% had HER2-negative tumors, with 21.3% of unknown status. Triple-negative (ER−, PR−, and HER2−) breast cancer carriers accounted for 11.9% (74) and luminal A tumors (ER or PR+, HER2−) for 29.7% (185).

### 2.2. Associations between Five SNPs and Breast Cancer Risk

In the present case-control study of 623 breast cancer patients and 620 cancer-free controls in a Chinese population, we genotyped five SNPs (rs8051542C/T, rs12443621A/G, rs3803662C/T, rs4784227C/T, and rs3112612T/C) in the *TOX3/LOC643714* gene using the SEQUENOM MassARRAY^®^ platform to test the hypothesis that these SNPs were associated with breast cancer risk in Chinese women. Of the five successfully genotyped SNPs, significant associations with breast cancer risk were found for rs4784227 and rs8051542, with ORs of 1.31 (95% CI, 1.10–1.57) and 1.26 (95% CI, 1.02–1.56) per T allele, respectively (Table 2). The two susceptibility loci (rs4784227 and rs8051542) showed a dose-dependent manner with a higher breast cancer risk among homozygous carriers than in heterozygous carriers (OR_homo_ = 1.51, 1.60; OR_heter_ = 1.42, 1.26, respectively; Table 2).

The T-rs4784227 allele exhibited significant associations with the status of three receptors (ER, PR, and HER2) in an additive model with the per-allele OR ranging from 1.39 to 1.51; the T-rs8051542 allele was associated with ER-positive and HER2-negative breast cancer carriers (*p* = 0.034, 0.051, respectively; Table 3).

The other three SNPs (rs12443621, rs3803662, and rs3112612) showed no significant associations with breast cancer risk in the entire data set (*P*_trend_ = 0.827, 0.826, and 0.850, respectively; Table 2). Likewise, no association of these SNPs was found by receptor status (data not shown).

### 2.3. Combined Effect of SNP rs4784227 and rs8051542 in TOX3/LOC643714

To determine whether women carrying more than one risk allele were at greater risk of breast cancer, we assessed the combined effect of the two significant SNPs (rs4784227 and rs8051542) in the additive and dominant models. The combined OR showed a significant stepwise increase, depending on the combined number of minor alleles present, with a maximum OR of 1.37 (95% CI, 1.09–1.72) in the additive model and 1.49 (95% CI, 1.14–1.95) in the dominant model (Table 4).

### 2.4. Linkage Disequilibrium of the SNPs in TOX3/LOC643714

**Figure 1.**
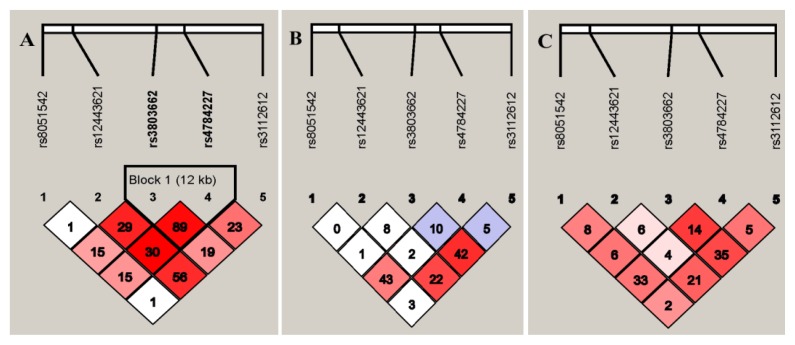
Linkage disequilibrium patterns among five SNPs in 16q12.1. The *r*^2^ values were presented; (**A**) the HapMap CEU population; (**B**) the HapMap CHB population; (**C**) Our data.

The five SNPs located in the *TOX3/LOC643714* region are contained in a 133-kb LD block [15,20]. According to previous reports [14,15,19], LD patterns differ between European and Asian descendants, which is consistent with our results (Figure 1). In the HapMap CEU population (which includes samples from Utah residents with northern and western European ancestry), SNP rs4784227, approximately 12.8 kb away from rs3803662, was in strong LD with rs3803662, with a Pearson’s correlation coefficient (*r*^2^) of 0.89. The *r*^2^ values were 0.15, 0.30, and 0.23 for rs8051542, rs12443621, and rs3112612, respectively (Figure 1). However, in the HapMap CHB population (which includes samples from Colorado residents of Chinese descent) and in our data, the five SNPs showed weak correlations with each other (Figure 1).

The five SNPs examined are located close to the *TOX3* and *LOC643714* genes. *LOC643714* is an uncharacterized gene of unknown function [16,21]. *TOX3*, also known as *TNRC9* (trinucleotide repeat containing 9), is located on 16q12.1 and is one of the low-penetrance breast cancer risk genes newly identified in GWAS [6]. It belongs to the large and diverse family of HMG-box proteins, which are nonhistone chromosomal proteins that bind to the minor groove in the DNA helix and are involved in chromatin structural modification [22]. *TOX3* has been identified as a calcium-dependent transactivator in neurons, and exerts its effect through interaction with both cAMP-response-element-binding protein (CREB) and the CREB-binding protein (CBP) complex [17,23]. Additionally, it can interact with CITED1 [23], a transcription co-activator that modulates the activity of transcription factors such as the ER [24] and SMAD4 [25]. However, the function of *TOX3* remains unclear.

Among five SNPs in the *TOX3/LOC643714* locus, we confirmed two breast cancer susceptibility loci (rs8051542 and rs4784227) among the southern Chinese population (*P*_trend_ = 0.030, *P*_trend_ = 0.003, respectively). Logistic regression analysis in the additive model indicated that the T-rs4784227 allele exhibited significant association with overall breast cancer, regardless of ER, PR, or HER2 receptor statuses. The T-rs8051542 allele was found to be associated with the risk of ER-positive and HER2-negative breast cancer. In contrast, none of the other three polymorphisms was significantly associated with breast cancer risk in either the whole data set or that stratified by the status of the three receptors.

SNP rs3803662 (C-to-T transition), which lies 8 kb upstream of *TOX3*, was one of significant variants associated with breast cancer risk identified through GWAS [6]; however, replication studies among European, African-American, and East Asian populations reported contradictory results [7,12,13,26,27]. Zheng *et al.* confirmed the significant association of both rs3803662 and rs8051542 and breast cancer risk in the genetic score contributing to the full risk assessment model, which showed promise for stratifying Asian women into different risk groups [15]. However, the T-rs3803662 allele was associated with a lower risk of breast cancer in a subgroup of African-American women, a result opposite of that in the other ethnic groups [7]. In addition, the association between rs3803662 with ER-status breast cancer carriers was inconclusive. Stacey *et al.* confirmed that the variant was associated more strongly with ER-positive than ER-negative disease [7]. A much larger study, including 12,974 ER-positive and 3,765 ER-negative cases, showed an association with both tumor subtypes [28]. In our present study, we failed to confirm an association between rs3803662 and breast cancer risk, and no evidence of associations with subtypes defined by ER, PR or HER2 status was found. Even with the previously clear association, our results for the T-rs3803662 allele suggested that identification of the causative variant remains problematic.

SNP rs8051542 was significantly associated with breast cancer risk, as evidenced by the 26% increased risk with the minor allele compared with the common allele. In addition, T-rs8051542 increased the risk of ER-positive and HER2-negative tumors. The association between rs8051542 and breast cancer was not found in a hospital-based Chinese population [29]. In another larger study, Long *et al.* reported that a borderline significantly increased risk was observed for the variant genotypes (CT/TT) of rs8051542, as well as an association with risk of ER-positive breast cancer in a Chinese population [13]. SNP rs12443621 showed no association with risk of all breast cancers or the subtype status of the three receptors, which is consistent with a report of Asian women [6]. In contrast to our results, the rs12443621 AG/GG genotypes were reported to be significantly associated with an increased risk of ER-positive breast cancer in a Chinese population [30], which could be due—at least in part—to the different LD patterns of the *TOX3/LOC643714* region in the different populations. In Europeans, moderate LD was observed for rs12443621-rs3803662 and rs12443621-rs4784227 (*r*^2^ = 0.29, 0.30, respectively). Conversely, no LD was observed among these SNPs in our data (*r*^2^ = 0.06, 0.04, respectively) or in the HapMap CHB population (*r*^2^ = 0.08, 0.02, respectively) (Figure 1).

A recent study showed that SNP rs3112612 had moderate predictive power for establishment of genetic risk models for early identification and optimal treatment of breast cancer in Ashkenazi Jewish women [31]; however, this SNP was not associated with breast cancer risk in our study.

The significant association was observed between rs4784227 and breast cancer risk, with an adjusted OR of 1.31 (95% CI, 1.10–1.57) per allele in the entire data set. Rs4784227 is also consistently significantly associated with breast cancer risk among European, Asian, and Korean women [6,19,26]; however, no evidence for an association with receptor status was reported by these studies. This SNP has been demonstrated to be a functional causal variant by the vast majority of breast-cancer-risk-associated SNP studies *in vitro* [19,32]. It was enriched in the cistromes of FOXA1 and ESR1, and the epigenome of histone H3 lysine 4 monomethylation (H3K4me1), and modulated the affinity of chromatin for FOXA1 at distal regulatory elements, resulting in allele-specific gene expression [32].

To date, the specific mechanism by which low-risk SNPs confer susceptibility to breast cancer risk is unclear. Evidence for *TOX3* as a breast cancer susceptibility gene is contradictory, which may account for the negligible effects of some polymorphisms on breast cancer risk. Furthermore, differences in the LD structure in the *TOX3/LOC643714* region between populations of European and Chinese origin are likely to play a role. Spanning a 133-kb LD block in a chromosomal region that exhibits evolutionary conservation, the five SNPs lie in the 5′ end of *TOX3* and near the last exon (exon 4) of *LOC643714* [20]. In the HapMap CEU population, rs4784227 showed strong LD with rs3803662, with an *r*^2^ of 0.89, while moderate correlations were observed with rs8051542, rs12443621, and rs3112612. However, in both the HapMap CHB population and our data, the five SNPs showed weak correlations with each other. Therefore, it is possible that the causal variant(s) tagged by SNP rs3803662 and/or rs4784227 in European ancestry populations would be tagged by SNP rs4784227 or rs8051542 in Chinese populations. Additionally, the number of identified SNPs was inadequate to explain the genetic variation across the *TOX3/LOC643714* locus. Ruiz-Narvaez *et al.* [12] identified four new genetic variants (rs3104746, rs3112562, rs3104793, and rs8046994) in the *LOC643714* gene, which may tag the same causal variant of breast cancer in African-ancestry populations instead of rs3803662, consistent with our results. We also provided the supplement data of LD structure between the four new SNPs and the five SNPs examined exhibiting moderate to low correlation in the HapMap CHB population, except for rs3112612-rs3104793 with high correlation (Figure S1). Therefore, further mapping and/or functional characterizations in diverse populations are warranted to determine the variants associated with breast cancer risk.

A limitation of this study was that it was not possible to investigate precise established risk factors (age at menarche, parity, age at menopause, hormone replacement therapy, family history, and body mass index) to detect gene-environment interaction effects. We did not collect these data. In addition, detailed tumor characteristics of *BRCA1/2* mutations were not available and so an interaction between *BRCA1/2* and the presence of the three receptors, which could explain the differences among carriers, could not be evaluated. Third, a potential selection bias could have been introduced because the control subjects were recruited from healthy volunteers who were excluded from immediate family history of cancer. However, the significant association of two identified SNPs with breast cancer remained after deleting the number of cases with family history of breast cancer or cancers (data not shown). Despite the limitations, a strength of our study was the substantial number of cases and controls, which significantly increased the statistical power. Additionally, we provided evidence for molecular etiologic heterogeneity by evaluating the association between SNP polymorphisms and receptor status.

## 3. Experimental Section

### 3.1. Subjects

The study was approved by the Nanfang Hospital Ethics Committee and all subjects provided written informed consent for clinical genetic testing and were anonymized for research studies prior to enrollment. 623 case patients and 620 control subjects included in the present case-control study were recruited from the outpatient and inpatient clinics of Nanfang Hospital, Southern Medical University, Guangzhou, Guangdong Province, China, from 2009 to 2010. Patients selected for the study were those with histologically confirmed breast cancer, which provided data regarding the ER, PR, and HER2 statuses of tumors. The control group comprised individuals without cancer and with no immediate family history of cancer.

### 3.2. DNA Extraction

Peripheral blood samples were drawn from all participants and stored at −70 °C until DNA extraction. Genomic DNA was extracted using a commercial blood DNA kit (TIANamp Genomic DNA Purification Kit; Tiangen Biotech, Beijing, China), according to the manufacturer’s instructions, and stored at −70 °C until use.

### 3.3. SNP Selection and Genotyping

We selected SNPs (rs8051542, rs12443621, rs3803662, rs4784227 and rs3112612) in the *TOX3/LOC643714* locus at 16q12.1 from the confirmatory results from GWAS or meta-analysis in multiple populations [6,7,11,20].

The five SNPs were genotyped using the SEQUENOM MassARRAY matrix-assisted laser desorption ionization-time of flight mass spectrometry platform (Sequenom, San Diego, CA, USA). Primers for multiplex PCR and extended reactions were designed using proprietary software (Assay Designer, version 3.1) provided by Sequenom Inc (San Diego, CA, USA). In accordance with the manufacturer’s instructions, SNPs were genotyped using Sequenom MassARRAY genotyping technology (Sequenom, San Diego, CA, USA) and amplified in multiplex PCR by a standard PCR protocol. The genomic amplification product was cleaned using shrimp alkaline phosphatase (Sequenom, San Diego, CA, USA) to neutralize any unincorporated dNTPs, followed by a single-base extension reaction using the iPLEX enzyme (Sequenom, San Diego, CA, USA) and mass-modified terminators (Sequenom, San Diego, CA, USA). The products of the iPLEX reaction were desalted and transferred onto a SpectroCHIP (Sequenom, San Diego, CA, USA) by the MassARRAY nanodispenser (Sequenom, San Diego, CA, USA), which was then analyzed by the MassARRAY analyzer by combining base calling with the clustering algorithm.

### 3.4. Statistical Analysis

Pearson’s χ^2^ analysis was used to test for independence of the alleles (HWE), selected variables, and frequencies of the genotypes of the five SNPs between cases and controls. All SNPs with a deviation from HWE in controls at *p* < 0.05 were excluded. The case-only *p* value was used to test for heterogeneity with receptor status. The associations between *TOX3* genotypes and the risk of breast cancer were modeled using an unconditional logistic regression model with two degrees of freedom (*i.e.*, codominant, dominant, and additive models) adjusted by age at diagnosis/selection. Odds ratios (OR) and 95% confidence intervals (CI) were calculated from these models for homozygote and heterozygote genotypes and per allele, which was used to test for a linear trend on a log scale in a dose-response relationship. We also performed case-control analyses by subgroups stratified according to ER/PR/HER2 status (comparing subtype cases to all controls). *p* < 0.05 was considered to indicate statistical significance. All statistical analyses were performed using SPSS version 13.0 (SPSS Inc., Chicago, IL, USA). Haplotypes were estimated using the web-based tool SNPstats (http://bioinfo.iconcologia.net/SNPStats) [33].

## 4. Conclusions

In the present study, our data suggest that *TOX3/LOC643714* rs8051542 and rs4784227 polymorphisms are significantly associated with the breast cancer risk. In addition, T-rs8051542 was significantly associated with ER-positive and HER2-negative tumors. However, no associations between rs12443621, rs3803662, and rs3112612 and risk of breast cancer were identified. Our results support the hypothesis that genetic factors differ according to ethnicity. Susceptibility loci common to various genetically admixed populations should be identified to explain the genetic variance in breast cancer risk and to improve our understanding of the complex biological mechanisms. Furthermore, it is necessary to conduct studies with larger sample sizes and that assess gene–gene and gene–environment interactions to establish powerful risk prediction models. This will reduce the incidence and mortality associated with breast cancer.

## Supplementary Information



## Figures and Tables

**Table 1.** Baseline characteristics of select variables in breast cancer cases (*n* = 623).

Variable	Value
Age, years (mean ± SD)	48.5 ± 10.0 (range 22–80)

Tumor histology (*n* = 559)	

IDC	524 (84.1%)
Others a	35 (5.6%)
Unknown	64 (10.3%)

Clinical staging of cancer (UICC) b (*n* = 559)	

Stage 0 (*in situ*)	6 (1.0%)
Stage 1	153 (24.6%)
Stage 2	154 (24.7%)
Stage 3	187 (30.0%)
Stage 4	59 (9.5%)
Unknown	64 (10.3%)

Receptor status	

Estrogen receptor (*n* = 517)	

Positive	300 (48.2%)
Negative	217 (34.8%)
Unknown	106 (17.0%)

Progesterone receptor (*n* = 516)	

Positive	267 (42.9%)
Negative	249 (40.0%)
Unknown	107 (17.2%)

Human epidermal growth factor receptor 2 (*n* = 490)	

Positive	230 (36.9%)
Negative	260 (41.7%)
Unknown	133 (21.3%)
Triple-negative c	74 (11.9%)
Luminal A d	185 (29.7%)

a mean ILC, Muc, Medul;

b mean International Union Against Cancer (UICC) stages;

c ER, PR, and HER2 all negative;

d ER or PR positive, HER2 negative.

**Table 2.** Association of breast cancer risk with five single nucleotide polymorphisms (SNPs) in *TOX3/LOC643714* in Chinese women.

SNP	Position	Alleles (reference/risk)	MAF (control/case)	Codominant model		Additive model	

				Heterozygote OR (95% CI) a	Homozygote OR (95% CI) a	Per-allele OR (95% CI) a	*P*_trend_ b
rs8051542	52534167	C/T	0.17/0.20	1.26 (0.98–1.62)	1.60 (0.83–3.10)	**1.26 (1.02**–**1.56)**	**0.030**
rs12443621	52548037	A/G	0.43/0.42	1.02 (0.74–1.40)	1.04 (0.74–1.45)	1.02 (0.86–1.20)	0.827
rs3803662	52586341	C/T	0.66/0.66	1.10 (0.76–1.59)	1.07 (0.74–1.56)	1.02 (0.86–1.21)	0.826
rs4784227	52599188	C/T	0.24/0.30	1.42 (1.12–1.81)	1.51 (0.97–2.35)	**1.31 (1.10**–**1.57)**	**0.003**
rs3112612	52635164	T/C	0.21/0.21	0.91 (0.71–1.16)	1.18 (0.68–2.04)	0.98 (0.81–1.19)	0.850

a OR adjusted for age;

b *P*_trend_ for per-allele;

Bold mean *p* < 0.05.

**Table 3.** Association of T-rs8051542 and T-rs4784227 with breast cancer subtypes in additive model.

Subtypes	T-rs8051542			T-rs4784227		
	
	Cases	OR (95% CI) a	*p* value	Cases	OR (95% CI) a	*p* value
Estrogen receptor (ER)						

ER-positive	596	**1.31 (1.02**–**1.69)**	**0.034**	598	**1.41 (1.13**–**1.75)**	**0.002**
ER-negative	430	1.18 (0.89–1.58)	0.246	434	**1.42 (1.11**–**1.81)**	**0.005**

Progesterone receptor (PR)						

PR-positive	530	1.40 (1.08–1.81)	0.100	534	**1.43 (1.14**–**1.79)**	**0.002**
PR-negative	494	1.10 (0.83–1.46)	0.486	484	**1.39 (1.10**–**1.75)**	**0.007**

Human epidermal growth factor receptor 2 (HER2)						

HER2-positive	456	1.24 (0.94–1.64)	0.131	458	**1.37 (1.08**–**1.75)**	**0.010**
HER2-negative	516	**1.30 (1.00**–**1.69)**	**0.051**	520	**1.51 (1.20**–**1.89)**	**0.000**

a OR adjusted for age;

Bold mean *p* < 0.05.

**Table 4.** Combined odds ratios for the two significant SNPs in *TOX3/LOC643714* in Chinese women.

Dominant model	rs8051542		Additive model	rs8051542	

rs4784227	CC	CTTT	rs4784227	C	T
CC	1.00	1.03 (0.59–1.77)	C	1.00	1.09 (0.68–1.73)
CTTT	1.36 (0.99–1.87)	**1.49 (1.14**–**1.95)**	T	1.29 (0.99–1.66)	**1.37 (1.09**–**1.72)**

Bold mean *p* < 0.05.
