# Supplementary material for: Risk-Association of Five SNPs in TOX3/LOC643714 with Breast Cancer in Southern China
Source: Int J Mol Sci. 2014 Jan 29;15(2):2130–41. doi: 10.3390/ijms15022130 (PMC3958841; doi:10.3390/ijms15022130)
Supplement: Supplementary file 1 [file ijms-15-02130-s001.pdf]

Supplementary Information

Figure S1. Linkage disequilibrium patterns among nine genotyped SNPs in *TOX3/LOC643714* in the HapMap CHB population.

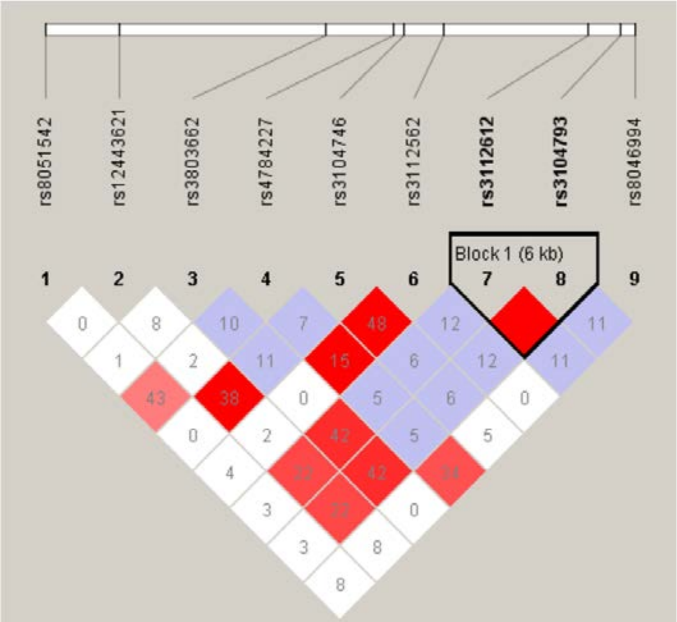

© 2014 by the authors; licensee MDPI, Basel, Switzerland. This article is an open access article distributed under the terms and conditions of the Creative Commons Attribution license (http://creativecommons.org/licenses/by/3.0/).
